# Ball Tracking and Trajectory Prediction for Table-Tennis Robots

**DOI:** 10.3390/s20020333

**Published:** 2020-01-07

**Authors:** Hsien-I Lin, Zhangguo Yu, Yi-Chen Huang

**Affiliations:** 1 Graduate Institute of Automation Technology, National Taipei University of Technology, No. 1, Sec. 3, Zhongxiao E. Rd., Taipei 10608, Taiwan; john6402j@gmail.com; 2 School of Mechatronical Engineering, Beijing Institute of Technology, Beijing 100081, China; yuzg@bit.edu.cn

**Keywords:** table-tennis robots, ball tracking and trajectory prediction, artificial neural networks

## Abstract

Sports robots have become a popular research topic in recent years. For table-tennis robots, ball tracking and trajectory prediction are the most important technologies. Several methods were developed in previous research efforts, and they can be divided into two categories: physical models and machine learning. The former use algorithms that consider gravity, air resistance, the Magnus effect, and elastic collision. However, estimating these external forces require high sampling frequencies that can only be achieved with high-efficiency imaging equipment. This study thus employed machine learning to learn the flight trajectories of ping-pong balls, which consist of two parabolic trajectories: one beginning at the serving point and ending at the landing point on the table, and the other beginning at the landing point and ending at the striking point of the robot. We established two artificial neural networks to learn these two trajectories. We conducted a simulation experiment using 200 real-world trajectories as training data. The mean errors of the proposed dual-network method and a single-network model were 39.6 mm and 42.9 mm, respectively. The results indicate that the prediction performance of the proposed dual-network method is better than that of the single-network approach. We also used the physical model to generate 330 trajectories for training and the simulation test results show that the trained model achieved a success rate of 97% out of 30 attempts, which was higher than the success rate of 70% obtained by the physical model. A physical experiment presented a mean error and standard deviation of 36.6 mm and 18.8 mm, respectively. The results also show that even without the time stamps, the proposed method maintains its prediction performance with the additional advantages of 15% fewer parameters in the overall network and 54% shorter training time.

## 1. Introduction

Recent years have seen the gradual maturing of sensory, machine vision, and control technology in smart robots. Several domestic and foreign studies explored the applicability of robots to sports. KUKA AG Robotics once made a commercial in which one of their robots played table tennis against a human, and Omron once gave a demonstration with one of their suspended robotic arms playing table tennis against a human at an automation exhibition. Table-tennis robots use a wide range of technologies, including object recognition, object tracking, 3D reconstruction, object trajectory prediction, robot motion planning, and system integration. This as well as the fact that they are easy to showcase attracted the attention of many researchers.

A ping-pong ball trajectory system combines vision, 3D space, and prediction algorithms, none of which are dispensable. The vision system must be able to detect and position the ball [1,2]. The data captured by cameras are two-dimensional (2D), so three-dimensional (3D) data cannot be derived by simply searching for the locations of object pixels. Wei et al. [3] proposed a method that uses a single camera to calculate object positions. In addition to using image recognition to locate the current pixels of the ball, they also used the shadow of the flying ball on the table to triangulate the spatial location of the ball. However, it is difficult to detect the shadow of a sphere, and the sources of light in general environments are complex and unpredictable. Their proposed approach was only useful if there was only a single clear light source in the environment with no sunlight or other light sources. The installation of two or more cameras can be used to establish stereopsis. Detection of an object from multiple perspectives can increase the dimensions of the image data and enable simple calculations of 3D data. Refs. [4,5] both adopted two cameras to track the ball in their vision system. To increase the visual coverage and accuracy of the vision system, Chen et al. [6] installed two high-speed cameras above the robot and above the opponent, which amounted to four cameras covering the entire table. Yang et al. [7] used six cameras to cover the table (three on each side) to achieve high precision for every possible location of the ball.

The processing speed of the vision system is another key factor because it indirectly affects prediction of the direction of the ball, particularly in prediction methods using physical models. Liu [8] proposed an onboard stereo camera system to estimate ping-pong ball trajectories in real time under the asynchronous observations from different cameras. Graphics processing units (plGPU) have become popular computer components in the recent trend of deep learning in image classification because they make the graphics card less dependent on the central processing unit (CPU) and improve the performance of graphics computing. Lampert et al. [9] employed an NVIDIA GeForce GTX280 graphics card and a CUDA framework to accelerate image processing and used four cameras to establish 3D space. Their system needed only 5 ms to process a 3D location. Furthermore, German company Nerian launched a real-time 3D stereo vision core that uses a field-programmable gate array (FPGA) as the hardware for parallel computation [10]. It can complete more tasks per clock cycle, send the computation results to a personal computer, and presents good performance in object recognition and 3D computation. Several studies used camera arrays to realize high-speed real-time object tracking and trajectory prediction. Zhang et al. [11] used a total of three cameras, one of which was a pan-tilt camera with a resolution of 640 × 480 pixels; its sampling frequency could reach less than 5 ms, and it could simultaneously follow a flying ball and analyze its status.

Accurate prediction of the ball trajectory is vital to the capacity of the robot to hit the ball. Existing prediction methods can be divided into two categories: physical models and machine learning. Physical models assess the external forces affecting the trajectory of the ball, such as constant gravity and air resistance. Balls with self-rotation are subject to a lateral force perpendicular to the plane formed by the angular velocity vector and the motion velocity vector of the rotation. This force, known as the Magnus effect, causes deflection in the flight trajectory of the ball. Ping-pong balls are not heavy, so the deflection is greater than that it would be with heavier objects [12,13]. Wang et al. [14] proposed a physical model to predict ball trajectories of topspin, backspin, rightward spin, leftward spin, and combined spin. Huang et al. [15] proposed a physical model that considers the self-rotation of the ball. It uses a real-time vision system to obtain a series of trajectory points and a camera system combining DSP and FPGA and fits the 3D data into a quadratic polynomial, which is then used to obtain the current flying velocity. In their experiments, their proposed method had good predictive abilities when the sampling time was 1 ms. However, if the consecutive trajectory points were not dense enough or if the sampling frequency was too low, the accuracy of current velocity estimates is affected. Inputting these estimates into complex formula calculations would then increase distortion in the final results. Zhang et al. [11] used two high-resolution cameras to analyze the self-rotation of the ball. Zhao et al. [16] used an ultrahigh-speed camera to analyze the collision between a ping-pong ball and the table and developed a physical model for self-rotation and collision effects. Ball status estimates can also be obtained using various filters, such a fuzzy filter [17], an extended Kalman filter [18], and an unscented Kalman filter [19]. Other studies presented analyses of the aerodynamics and the friction between ping-pong balls and tables [20,21].

Machine learning has also tended towards table-tennis research in recent years. Some of the table-tennis robots developed in these studies employed locally weighted regression algorithms to learn hitting movements [22,23]. In machine learning algorithms, machines analyze data to find regularities and then use the regularities to make predictions of unknown data. Zhao et al. formulated the likelihood of ping-pong ball motion state as a Gaussian Mixture Model (GMM) [24]. Deng et al. [25] proposed a ball-size likelihood to estimate the ball position. Payeur et al. [26] used an artificial neural network (ANN) to learn and predict the trajectories of moving objects; however, they merely performed simulation experiments of simple trajectories and did not develop a novel vision system or robot. Nakashima et al. Nakashima et al. [27] used a back-propagation artificial neural network (BPANN) to learn striking points. The inputs are the initial location and velocity difference of the ball in a free-fall model, and the outputs are the striking point and displacement between striking points estimated using simple physics. Although their simulation results were good, the model requires 20 trajectory points for fitting, which implies that the sampling frequency of the vision system must be high. Zhang et al. [3] also made an attempt with four initial trajectory points and time stamps as the inputs of the network and the location and velocity of the striking point as the outputs.

Our objective is to track and predict ping-pong ball trajectories for a robot to hit the ball. Due to the facts that physical models for ball flight prediction require advanced vision equipment and that most physical models are fairly complex, we adopted machine learning to achieve prediction of ping-pong ball trajectories with limited equipment. To achieve better prediction, this study included a ball tracking system, 3D reconstruction, and ball trajectory prediction. The novelty of this work is that the flight trajectory between the serving point and the end of the robotic arm was viewed as two parabolas and two separate ANNs were used to learn these two parabolas. The ball trajectory prediction strategy proposed in this study makes the following contributions:

- We propose an ANN-based approach to learn historical trajectory data, thereby doing away with the need for a complex flight trajectory model.
- The proposed method can swiftly predict where the robot should strike the ball based on the ball location data from only a few instants after it is served, thereby giving the robot time to react.
- The inputs of ANNs are generally accompanied by time stamp data. We verified that removing the time stamp data reduces the parameter demand of the entire network and greatly shortens network training time.

**Figure 1:**
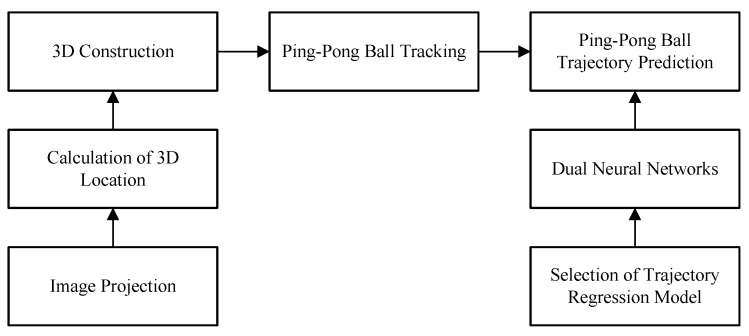
Flow of the proposed method.

Figure 1 shows the flow of the proposed method. There are three main parts: 3D construction, ping-pong ball tracking, and ping-pong trajectory prediction. 3D construction and ping-pong ball tracking are used to obtain the accurate ball current position. To hit the ball, we propose the dual neural networks to predict the ball position on the hitting plane. These three parts are explained in Section 2.

The remainder of this paper is organized as follows. Section 2 introduces the framework for 3D reconstruction, and Section 3 explains the ball trajectory prediction method proposed in this study. Section 4 presents the experiment results, and Section 5 contains our conclusion.

## 2. 3D Reconstruction Framework

### 2.1. Hardware Setup

**Figure 2:**
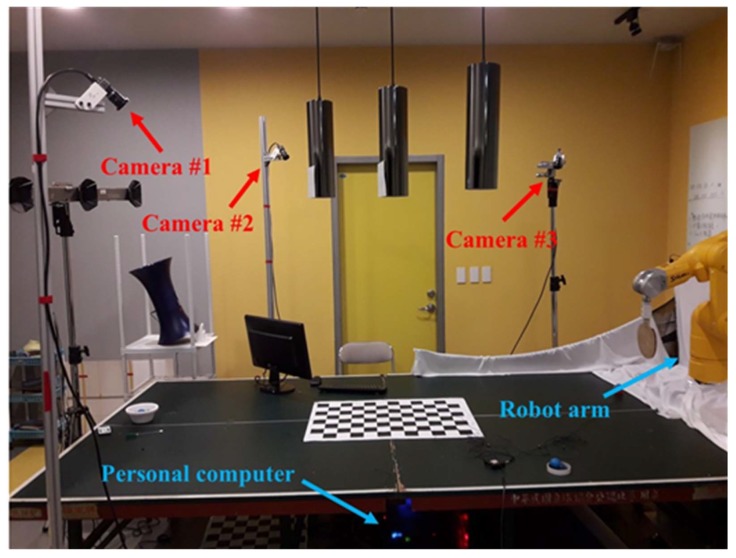
3D reconstruction system of table-tennis robot.

Figure 2 displays the 3D space system of this study. The vision system comprises three IDS color industrial cameras with 1.3 megapixel resolution. The FPS of the cameras was set at 169, and each had to cover most of the table surface within their field of view. The cameras were placed on both sides of the table: one on the right (camera#1), one on the left (camera#2), and an auxiliary camera (camera#3). The farthest visible distance of the camera system was approximately 220 cm to facilitate the widest range of tracking. In addition, we noted whether the color of the background would interfere with tracking. The table-tennis robot was a Stäubli industrial robot arm, which has six degrees of freedom, repeatability of ±0.02 mm, a reach of 670 mm, and a maximum speed of 8.0 m/s at the endpoint. We used the high-precision Phoenix Technologies Inc. VZ4000v 3D motion capture system, which can achieve an accuracy of 0.015 mm within a distance of 1.2 m, to analyze 3D errors in the images.

**Figure 3:**
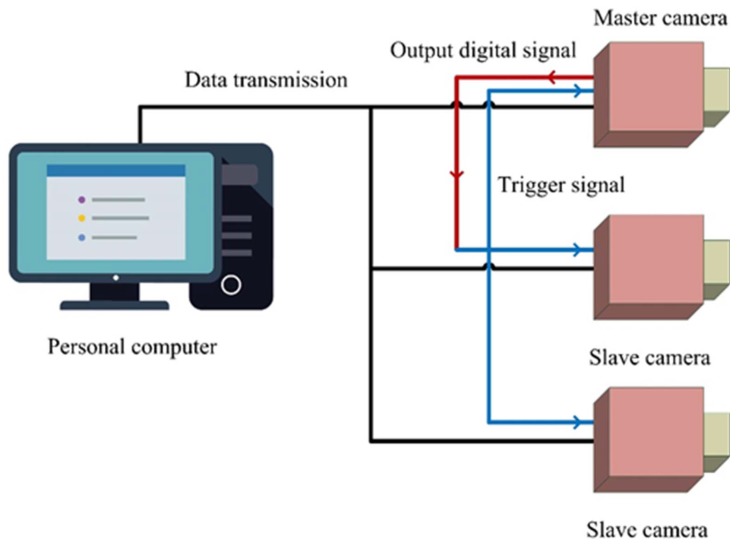
Synchronization procedure of the multi-camera vision system.

Camera synchronization is an important issue in the multi-camera vision system. When computer CPUs use multithreading, the various threads may not be executed at the same time depending on the resource allocation decisions of the CPU at the time. Thus, the timing at which the cameras capture images may not be the same. We used a master camera to send image capture signals. The slave cameras as well as the master camera wait for the signal. Figure 3 shows the synchronization process of the camera system. To validate the synchronization process, we collected one hundred trajectories with synchronized and unsynchronized procedures. Curve fitting was performed for each trajectory, and then the mean-square error was analyzed. The mean-square errors with synchronized and unsynchronized procedures were 7.7 mm and 12.1 mm, respectively. The result validated that the synchronization procedure helped the multi-camera vision system to acquire synchronized images.

**Figure 4:**
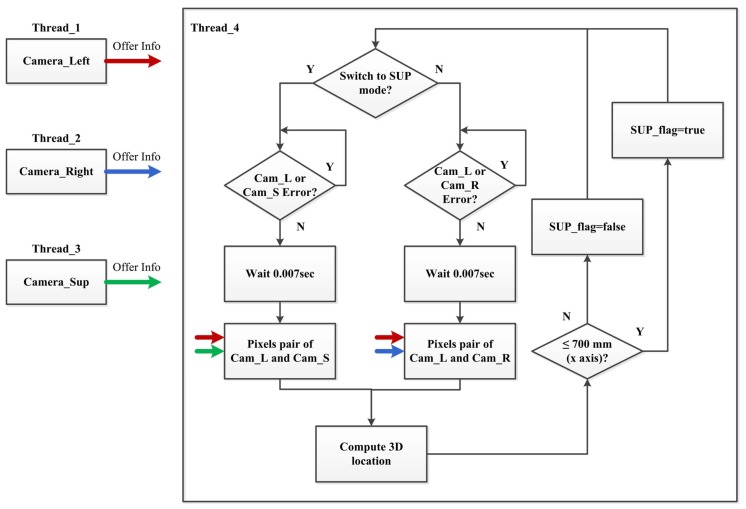
Control of the cameras.

Figure 4 shows the control of the cameras with the sampling time of 0.007 s. Threads 1 to 3 are the image processing flow for each of the three cameras. When the ball is getting away from camera#1 and camera#2, the 3D position error will increase. Therefore, the auxiliary camera#3 is used to reduce the error. The timing of using camera#3 is when the *x*-axis position of the ball is greater than 700 mm (the robot side is the origin), camera#1 and camera#2 are used; otherwise, camera#1 or camera#2 is used with the auxiliary camera#3 depending on whether the ball falls to the left or right of the table.

### 2.2. 3D Reconstruction

We used two industrial cameras to establish a 3D space. Using the pixel locations of the target from two perspectives, we positioned the location of the target in the 3D space. The coordinate system of the cameras was then converted into the coordinate system of the robot arm. In this way, deriving the 3D location of an object in images from the camera system would also give us the location of the object in the 3D space. This approach included camera calibration and triangulation. However, the error increases when the object is further away from the camera system. This is because the pixel features of objects further away are not as clear and the pixel resolution at this distance has reached its limits. We will give a complete explanation in Section 2.4.

### 2.3. Ping-Pong Ball Tracking

To track the ball, the field of view of the cameras must encompass the entire table, which includes various complex colors and noise. To simplify image segmentation, we painted the ping-pong ball blue to distinguish other objects and the background. A Gaussian blur is first applied to the images captured by the cameras to remove noise and facilitate the subsequent recognition process. HSV color space conversion is then used to reduce the impact of bright light sources, and then a threshold value is easily set for the color of the ball to obtain a binary image. To make the target object more complete, morphological operators erosion and dilation are applied and then the median values of the binary image are calculated to serve as the ball position. Performing these processes on images with large fields of view is time-consuming. To save time, we employed the region-of-interest (ROI) operation. Once the camera tracked the ball, then only the ROI in the images is processed. The FPS was set at 169 in this study. When an entire image with 1280 × 1024 pixels is subjected to the object recognition procedure, the average frequency is approximately 50.7 times/s. If an ROI is adopted, then subjecting the ROI (an image of 200 × 200 pixels) to object recognition results in an estimated frequency of 514.5 times/s. Clearly, the ROI mechanism can significantly increase the efficiency of object recognition.

#### 2.3.1. Image Projection

**Figure 5:**
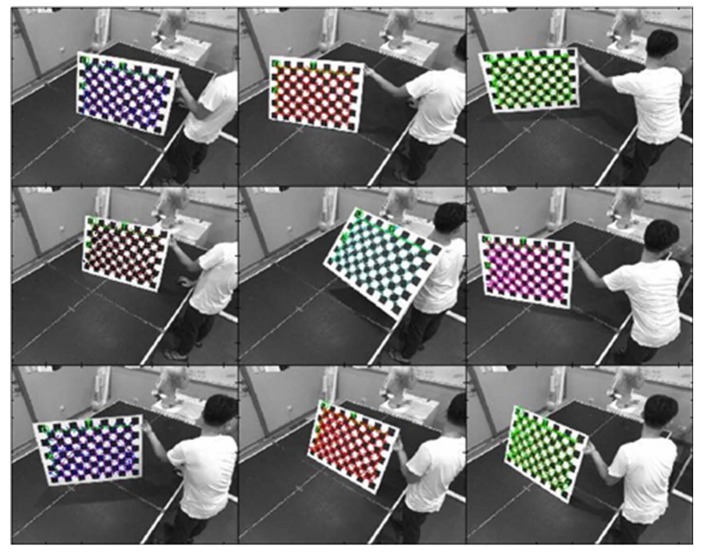
Calibration using checkerboard at different positions.

The camera calibration was to obtain pixel scale, intrinsic matrix, and extrinsic matrix of the camera. Figure 5 shows that camera calibration was conducted using the checkerboard data from 30 images of a 13 × 9 checkerboard with 60 × 60 mm^2^ at various angles, depths, and locations. Table 1, Table 2, Table 3 and Table 4 present the intrinsic and extrinsic parameters of the right and left cameras.

#### 2.3.2. Calculation of 3D Location

**Figure 6:**
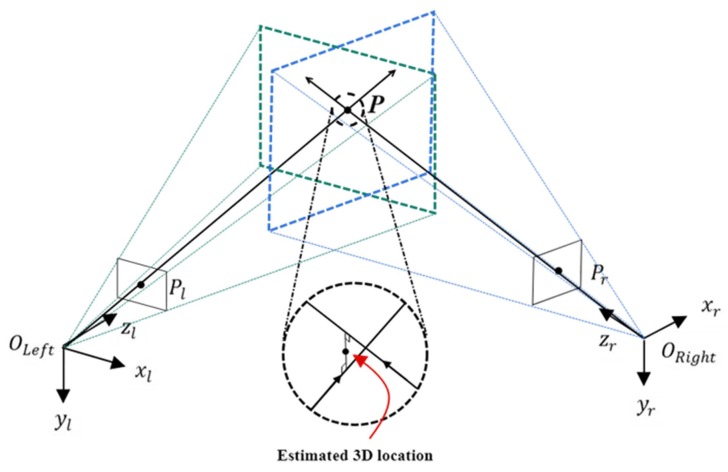
Calculation of 3D location.

As shown in Figure 6, the location of the target object can be obtained when it is within view of the two cameras. *P* is the location of the object, and $O_{Left}$ and $O_{Right}$ denote the respective origins of the coordinate systems of the left and right cameras. $P_{l}$ and $P_{r}$ represent the pixel locations of the object in the images taken by the left and right cameras. Figure 6 shows that the plane formed by $O_{Left}$, $O_{Right}$, and *P* is defined as an epipolar plane. This characteristic can be used to identify the physical relationship between the two cameras [28]. Another approach is to assume that $M_{w}^{l}$ and $M_{w}^{r}$ are homogeneous matrices converting world coordinates to left and right camera coordinates using the extrinsic parameters of the cameras, respectively. With $M_{w}^{r}$ as an example, formula calculations produce $M_{r}^{w}$, as shown in Equation (1). Multiplying $M_{w}^{l}$ by $M_{r}^{w}$ then gives $M_{r}^{l}$, as shown in Equation (2). This is the rotation and translation matrix converting the coordinates in the right camera system to those in the left camera system.

(1) $$\begin{array}{c} M_{r}^{w}=\left[\begin{array}{cc} {\left(R_{w}^{r}\right)}^{T} & -{\left(R_{w}^{r}\right)}^{T}\left(T_{w}^{r}\right)\\ 0_{1X3} & 1 \end{array}\right] \end{array}$$

(2) $$\begin{array}{c} \left(M_{l}^{r}\right)=\left(M_{l}^{w}\right)\left(M_{r}^{w}\right) \end{array}$$

Figure 6 shows the centers of the left and right cameras both pointed at target *P*. The vectors pointing from the centers of the two cameras to the pixel location of the target object are defined as ${\tilde{P}}_{l}$ and ${\tilde{P}}_{r}$, as shown below:

(3) $$\begin{array}{c} {\tilde{P}}_{l}=\left[\begin{array}{c} \left(x_{l}^{\prime }-c_{lx}\right)s_{lx}\\ \left(y_{l}^{\prime }-c_{ly}\right)s_{ly}\\ f_{l} \end{array}\right] \end{array}$$

(4) $$\begin{array}{c} {\tilde{P}}_{r}=\left[\begin{array}{c} \left(x_{r}^{\prime }-c_{rx}\right)s_{rx}\\ \left(y_{r}^{\prime }-c_{ry}\right)s_{ry}\\ f_{r} \end{array}\right] \end{array}$$

where $x_{l}^{\prime }$ and $y_{l}^{\prime }$ is the pixel location of the object in the left image; $c_{lx}$ and $c_{ly}$ is the image center of the left camera; $s_{lx}$ and $s_{ly}$ are the scale coefficients of the left camera; $x_{r}^{\prime }$ and $y_{r}^{\prime }$ is the pixel location of the object in the right image; $c_{rx}$ and $c_{ry}$ is the image center of the right camera, and $s_{rx}$ and $s_{ry}$ are the scale coefficients of the right camera.

**Figure 7:**
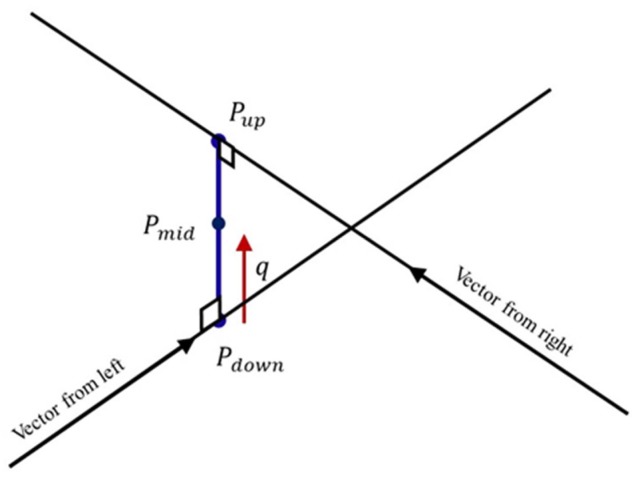
Two vectors in a skew relationship.

In the 3D space, these two vectors intersect at the location of the target object. However, this only occurs in ideal circumstances. Figure 7 displays the more likely circumstance in which the two vectors are skew and do not intersect. Thus, we assume that the target object is located at the middle point of the line segment that is the shortest distance between the two vectors. Based on rigid body transformation, vector ${\tilde{P}}_{r}$ of the right camera can be converted into a vector with regard to the left camera system using $M_{r}^{l}$, the physical relationship between the two cameras, which is $R_{r}^{l}$ and $T_{r}^{l}$. Equation (5) represents the distance to which the vector should extend to reach $P_{up}$. Here, we assume there is an unknown coefficient *b*. Similarly, Equation (6) represents the distance to which the left vector should extend to reach $P_{down}$, where we assume there is an unknown coefficient *a*.

(5) $$\begin{array}{c} P_{up}=\left(R_{r}^{l}\right)b{\tilde{P}}_{r}+T_{r}^{l} \end{array}$$

(6) $$\begin{array}{c} P_{down}=a{\tilde{P}}_{l} \end{array}$$

To derive $P_{mid}$, we must calculate the directional vector *q* of the line segment. This vector can be obtained using the cross product of the left and right vectors, as shown in Equation (7). Please note that ${\tilde{P}}_{r}$ must also undergo coordinate rotation conversion $R_{r}^{l}$, and the unit vector of *q* is $\frac{q}{\left|q\right|}$.

(7) $$\begin{array}{c} q={\tilde{P}}_{l}\times \left(R_{r}^{l}{\tilde{P}}_{r}\right) \end{array}$$

The length of the line segment is unknown, so we suppose that *c* is the coefficient of the unit vector of *q*. Based on basic concepts of vectors, this line segment can be expressed as follows:

(8) $$\begin{array}{c} P_{up}=P_{down}+c\frac{q}{\left|q\right|} \end{array}$$

Further derivation gives

(9) $$\begin{array}{c} a{\tilde{P}}_{l}+\frac{c({\tilde{P}}_{l}\times R_{r}^{l}{\tilde{P}}_{r})}{|{\tilde{P}}_{l}\times R_{r}^{l}{\tilde{P}}_{r}|}=b\left(R_{r}^{l}\right){\tilde{P}}_{r}+T_{r}^{l} \end{array}$$

Suppose that

$$\begin{array}{c} A=\left[\begin{array}{c} {\tilde{P}}_{l}\;-R_{r}^{l}{\tilde{P}}_{r}\;{\frac{q}{\left|q\right|}}_{3\times 3} \end{array}\right] \end{array}$$

Equation (9) can be simplified into Equation (10):

(10) $$\begin{array}{c} \left[\begin{array}{c} a\\ b\\ c \end{array}\right]=A^{-1}T_{r}^{l}. \end{array}$$

We can then derive coefficients *a*, *b*, and *c*, and use *a* and *c* to calculate $P_{mid}$, as shown in Equation (12):

(11) $$\begin{array}{c} P_{mid}=a{\tilde{P}}_{l}+\frac{1}{2}\left(c\frac{q}{\left|q\right|}\right) \end{array}$$

Here, $P_{mid}$ is the 3D location of the target object with regard to the left camera coordinate system. Using $M_{l}^{w}$, we can convert the coordinates from the left camera system to the world coordinate system, as shown in Equation (12). This matrix can be reversely obtained using extrinsic parameter $M_{w}^{l}$ of the left camera.

(12) $$\begin{array}{c} P^{w}=M_{l}^{w}P_{mid} \end{array}$$

### 2.4. Estimation Error Analysis

**Figure 8:**
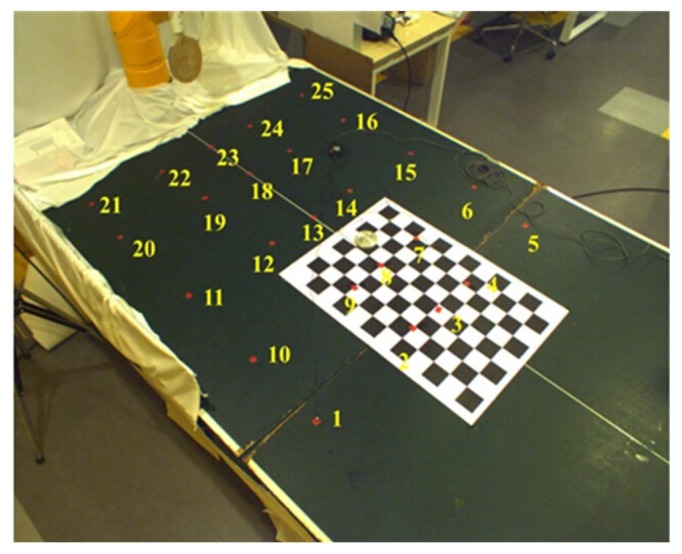
Locations of 25 trackers.

**Figure 9:**
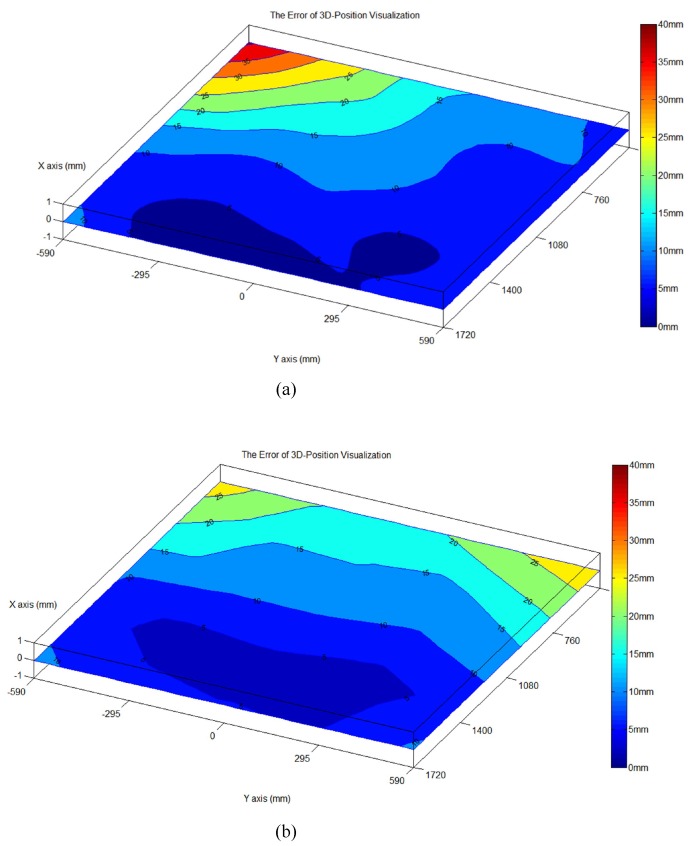
Distributions of the errors (**a**) using the right camera (camera#1) and the left camera (camera#2); (**b**) using the left camera (camera#2) and the auxiliary camera (camera#3).

Even using the algorithm above to calculate the 3D location of the target object, errors still exist in the data, and they increase with the distance between the target object and the origins of the camera coordinate systems. This section analyzes the errors in the use of the camera systems to calculate the locations of the target object. Here, we used a PTI motion capture system to serve as the control group for the 3D locations obtained using the three cameras. Figure 8 exhibits the 25 trackers that we used. The world coordinates of the PTI motion capture system overlapped those of the camera systems, meaning that the camera systems and the PTI motion capture system had the same world coordinates. To calculate the errors, we employed two stereopsis groups, one using the right camera (camera#1) and the left camera (camera#2) to calculate the 3D location of the PTI tracker and the other using the left camera (camera#2) and the auxiliary camera (camera#3). Using cubic interpolation, we derived a new error distribution graph. Figure 9 displays the distributions of the errors interpolated using cubic polynomials in the two stereopsis groups. As can be seen, the errors increase with the distances between the left and right cameras and the trackers and is highest at Tracker No. 21, where the error is 40 mm. With the left camera and the auxiliary camera, the two cameras are closer to Tracker No. 21, so the error reduces to approximately 25 mm.

## 3. Ping-Pong Ball Trajectory Prediction

If a table-tennis robot wants to hit the ball, it must be able to accurately predict where the ball is flying. When people play table tennis, the ball is subjected to various external forces that impact its flight direction and velocity, including air resistance, the Magnus effect, gravity, and the rebound force of collision. Thus, calculating the various physical forces with precision and then predicting the flight trajectory is difficult. In this section, we present a machine learning approach that enables robots to predict trajectories. In this section, we demonstrate that the coefficients of a polynomial regression model are advantageous to represent and predict ball trajectories.

**Figure 10:**
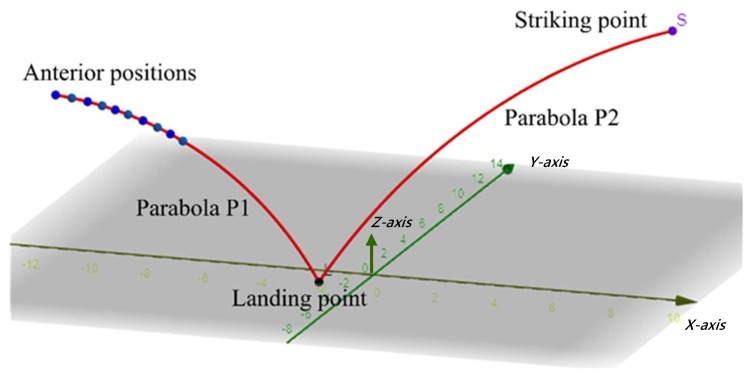
Diagram of trajectory prediction.

Figure 10 shows the flight trajectories of a ping-pong ball. The flight trajectories are divided into two parabolas P1 and P2. The first ANN is responsible for learning the parabola P1 in the figure, with the anterior positions as the input and the regression coefficient of P1 as the output. The second ANN learns parabola P2, with the regression coefficient of P1 as the input and the regression coefficient of P2 as the output. The trajectory coefficients above are the parameters of their mathematical expressions. Once the vision system detects the anterior positions, the system will immediately derive the coefficient P2 of the trajectory after the ball’s landing point on the table and then calculate a designated striking point. Suppose $f_{x}$, $f_{y}$, and $f_{z}$ denote the mathematical regression formulas of trajectory P2 along the *x*, *y*, and *z* axes. Using $f_{x}\left(t\right)$, the timing *t* of the designated striking point (*x* axis) is first derived. The resulting *t* is then substituted into $f_{y}$ and $f_{z}$ to obtain the *y* and *z* coordinates of the striking point.

Since the regression model should be chosen to predict the ball trajectory, we evaluate several models such as exponential, Fourier, Gaussian, and polynomial curve fitting methods in Section 3.1. The result shows that polynomial curve fitting is the most suitable model to predict the ball trajectory. In Section 3.2, we also theoretically verify the existence of the relationship between the polynomial coefficients and ball trajectory. However, Section 3.2 shows that curve fitting is highly sensitive to noise in the input data. Thus, we propose two ANNs to model the relationship in Section 3.4.

### 3.1. Selection of Trajectory Regression Model

The data of any parabolic trajectory include time stamps and location data. Thus, we must decide which type of regression model to use to express the trajectory data. This process mainly involves finding the relationship between a set of independent and dependent variables and then establishing an appropriate mathematical equation, which is referred to as a regression model. Below, we investigate which type of regression model is more suitable for the trajectory data.

**Figure 11:**
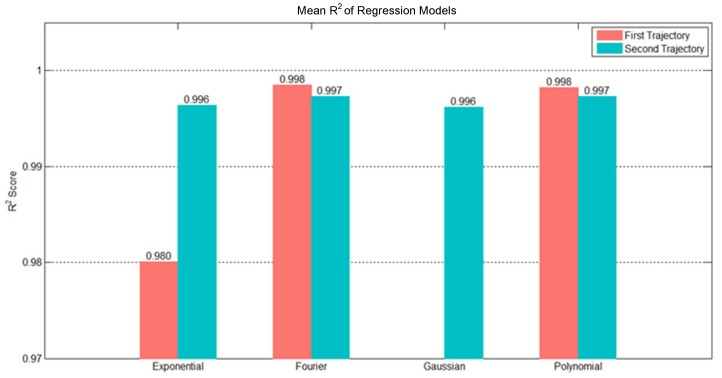
Diagram of trajectory prediction.

**Figure 12:**
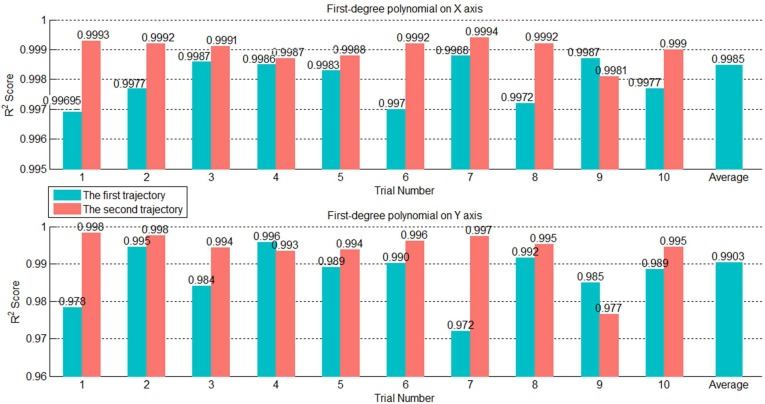
$R^{2}$ of the first-order polynomial regression on the *X* and *Y* axes.

We can use experiment data to determine a minimal number of parameters of suitable regression models. The experiment data comprised 10 random trajectories selected from the training data. Each flight trajectory consists of two parabolic trajectories: the first from the initial point to the landing point and the second from the landing point to the end point. We fitted the two trajectories using four types of regression models, namely, Gaussian, exponential, Fourier, and polynomial, and examined their results. We selected 10 random trajectories from the testing data to test $R^{2}$ results of these models. The red and blue bars in Figure 11 indicate the mean $R^{2}$ results. The Fourier and polynomial regression models presented reasonable $R^{2}$ results for both trajectories, and their test results were close to each other. However, the polynomial regression model used three parameters, whereas the Fourier regression model used four parameters. With our objective of minimizing the number of parameters, we ultimately chose the polynomial regression model to express the flight trajectory data. The polynomial regression model for the Z axis used three parameters, which was the quadratic polynomial. For the *X* and *Y* axes, Figure 12 shows $R^{2}$ of each trajectory and the average $R^{2}$ of the 10 random trajectories was 0.9903 using the first-order polynomial, which was close to 1. Thus, the *X* and *Y* axes were represented by the first-order polynomial regression.

### 3.2. Theoretical Verification of Trajectory and Polynomial Coefficients

When the two parts of the ping-pong ball trajectory can be fitted using a polynomial, it means that the coefficients in the polynomial effectively express the trajectory. We could therefore use ANNs to predict the polynomial coefficients of the ball trajectories. The polynomial coefficients and conversion relations between trajectories can be expressed using Equation (13). Suppose that ($t_{k}$,$q_{k}$) is several sets of data points where k=0,…,m, $t_{k}$ is the time value, and $q_{k}$ is the positional value. Here there exists a unique polynomial q(t) using the $n_{th}$ order. Equation (14) establishes the relation matrix pf vectors *q* and *a*, which is known as the Vandermonde matrix *T* [29]. Using the pseudo inverse matrix of *T*, Equation (15) gives coefficient a where a minimum squared-error exists between the coefficient equation and the trajectory data.

(13) $$\begin{array}{c} q\left(t\right)=a_{0}+a_{1}t^{1}+\cdots +a_{n}t^{n} \end{array}$$

(14) $$\begin{array}{c} q=\left[\begin{array}{c} q_{0}\\ q_{1}\\ .\\ .\\ .\\ q_{m-1}\\ q_{m} \end{array}\right]=\left[\begin{array}{c} 1\;t_{0}^{1}\;\cdots \;t_{0}^{n}\\ 1\;t_{1}^{1}\;\cdots \;t_{1}^{n}\\ .\\ .\\ .\\ 1\;t_{m}^{1}\;\cdots \;t_{m}^{n} \end{array}\right]\left[\begin{array}{c} a_{0}\\ a_{1}\\ .\\ .\\ .\\ a_{n-1}\\ a_{n} \end{array}\right]=Ta \end{array}$$

(15) $$\begin{array}{c} a=T^{-1}q \end{array}$$

The input and output data of ANNs must be interrelated in a certain way; otherwise, training the model may be too time-consuming, or poor prediction results may be produced. To verify that the use of ANNs to learn trajectories in this study is appropriate. Based on this mathematical theory, the trajectory point data can be mapped onto polynomial coefficient data using the Vandermonde matrix. Thus, a corresponding relationship exists between the elements of these two sets of data. When the trajectory points serve as the inputs of the ANN and the trajectory coefficients are the outputs, then the model has good learning ability.

### 3.3. Comparison of Artificial Neural Network and Polynomial Curve Fitting Predictions

**Figure 13:**
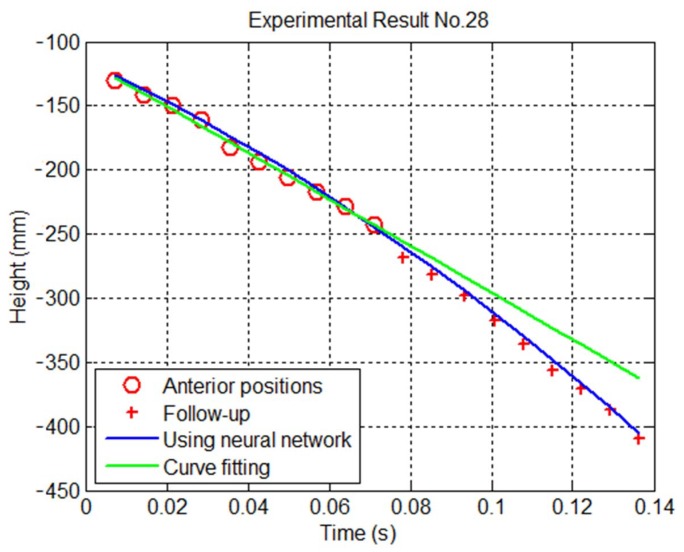
Trajectory based on ten positions (Blue: ANN; green: quadratic polynomial resulting from curve fitting).

In the prediction procedure of this study, once the vision system receives the data from a few anterior positions, the ANN of the first trajectory can then predict the first trajectory. To demonstrate the inadequacy of curve fitting and the prediction performance of the ANN for the first trajectory, we predicted the landing point of the first trajectory using the proposed method and a quadratic polynomial resulting from curve fitting. We used 30 items of data to train the ANN for the first trajectory and used the trained ANN and the ten anterior positions in the trajectory to predict the polynomial parameters of the first trajectory. The red circles and crosses in Figure 13 show the trajectory obtained during testing. The blue curve presents the polynomial trajectory output by the trained ANN, and the green curve shows the subsequent trajectory trend after the data from the ten anterior positions were received. The mean error and standard deviation of the quadratic polynomial were 19.2 mm and 11.8 mm, respectively; however, for the ANN, the mean error and standard deviation of the quadratic polynomial were 10.8 mm and 10.0 mm, respectively. The errors in the landing point predictions of the ANN were smaller than those of the quadratic polynomial. Once some errors exist in capturing the ten anterior positions of the ball, Figure 13 shows that the ANN is resistant to noise; even if an input node contains noise or its data is incomplete, the single data does not greatly impact the overall result.

### 3.4. Two ANNs in Trajectory Prediction

**Figure 14:**
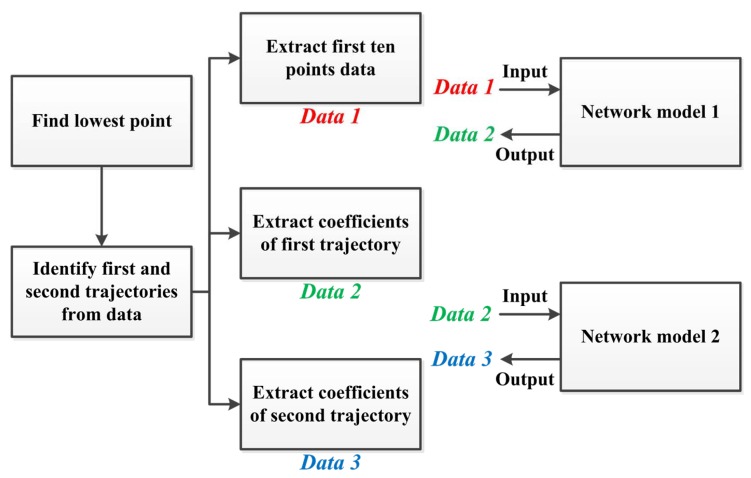
Procedure of generating the required training data.

Three things can be obtained from a complete flight trajectory: the trajectory before landing, the trajectory after landing, and ten anterior positions. Figure 14 displays the procedure of generating the required training data. First, the lowest point in the overall trajectory along the *z* axis is identified, demarcating the data of the first and second trajectory. The first trajectory is between the initial point and the lowest point, and the second trajectory is between the lowest point and the end of the trajectory. Both trajectories are then expressed using polynomial regression to extract the trajectory parameters and the data of the ten anterior positions. The data of the ten anterior positions and the parameters of the first trajectory then serve as the training data for the first ANN (Network Model 1), and the parameters of the first and second trajectories serve as the training data for the second ANN (Network Model 2).

We defined the 3D polynomial of the first trajectory as Equation (18), where $a_{1}$, $b_{1}$, $a_{2}$, $b_{2}$, $a_{3}$, $b_{3}$, $c_{3}$ are the *X*-, *Y*-, and *Z*-axis coefficients of the first trajectory. We defined the 3D polynomial of the second trajectory as Equation (21), where $a_{1}^{\prime }$, $b_{1}^{\prime }$, $a_{2}^{\prime }$, $b_{2}^{\prime }$, $a_{3}^{\prime }$, $b_{3}^{\prime }$, $c_{3}^{\prime }$ are the *X*-, *Y*-, and *Z*-axis coefficients of the second trajectory. Equations (22) and (23) are the corresponding inputs and outputs of the two networks.

(16) $$\begin{array}{ccc} X & = & a_{1}t+b_{1} \end{array}$$

(17) $$\begin{array}{ccc} Y & = & a_{2}t+b_{2} \end{array}$$

(18) $$\begin{array}{ccc} Z & = & a_{3}t^{2}+b_{3}t+c_{3} \end{array}$$

(19) $$\begin{array}{ccc} X & = & a_{1}^{\prime }t+b_{1}^{\prime } \end{array}$$

(20) $$\begin{array}{ccc} Y & = & a_{2}^{\prime }t+b_{2}^{\prime } \end{array}$$

(21) $$\begin{array}{ccc} Z & = & a_{3}^{\prime }t^{2}+b_{3}^{\prime }t+c_{3}^{\prime } \end{array}$$

(22) $$\begin{array}{c} {\left[P_{i},t_{i}\right]}_{i=1,\ldots ,10}\;\underset{\to }{\;\;\mathrm{Network}\mathrm{model}1\;\;}\;\left[a_{1},b_{1},a_{2},b_{2},a_{3},b_{3},c_{3}\right] \end{array}$$

(23) $$\begin{array}{c} \left[a_{1},b_{1},a_{2},b_{2},a_{3},b_{3},c_{3}\right]\;\underset{\to }{\;\;\mathrm{Network}\mathrm{model}1\;\;}\;\left[a_{1}^{\prime },b_{1}^{\prime },a_{2}^{\prime },b_{2}^{\prime },a_{3}^{\prime },b_{3}^{\prime },c_{3}^{\prime }\right] \end{array}$$

## 4. Experimental Results

**Figure 15:**
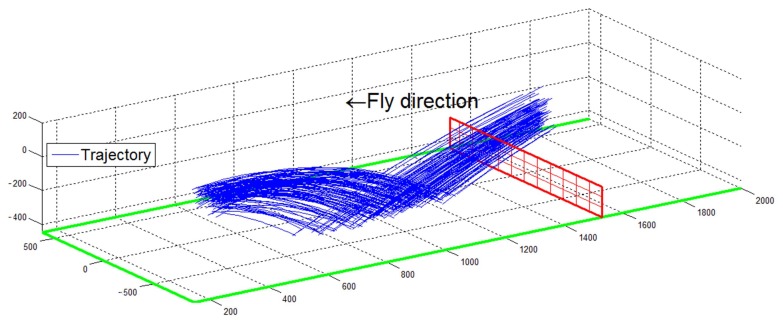
Flight trajectory data collected from human demonstrations.

In this section, we present a series of experiments conducted to demonstrate the prediction performance of the proposed method. In Section 4.1, we compare the striking point prediction performance of the proposed dual-network method and a single ANN [3] using the experimental data. In Section 4.2, we compare the striking point prediction performance of the proposed dual-network method and a physical model. Section 4.3 presents an experiment investigating the influence of time data removal, and Section 4.4 analyzes the errors of the proposed method in a physical experiment. Figure 15 shows the flight trajectory data collected from human demonstrations, in which the ping-pong ball was hit from the serving side to a robot arm on the opposite side. The first trajectories passed over the net and landed on the table on the robot’s side. We collected data from a total of 200 trajectories, among which 170 trajectories were used as training data and 30 trajectories comprised the testing data. Once the ANNs were trained, we used the testing data to examine their predictive abilities. We used a circle with a diameter of 75 mm to simulate the area of the paddle and calculated the errors, standard deviation, the number of balls that hit the range of the paddle, and the success rates.

### 4.1. Dual-Network vs. Single-Network Approach

**Figure 16:**
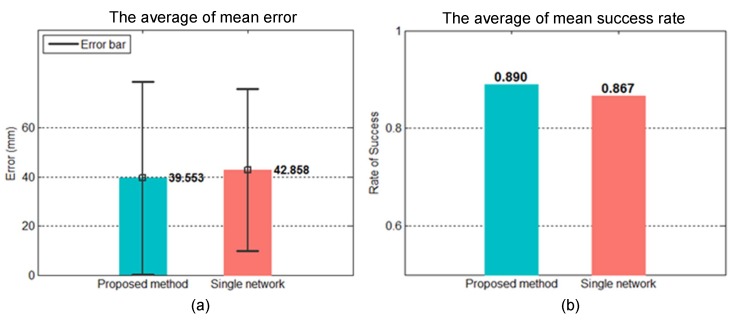
Performance results of proposed dual-network method and single-network approach: (**a**) mean error; (**b**) mean success rate.

Reference [3] used a single ANN to predict the striking points. The inputs were the data of four anterior positions, and the outputs were the striking point and the acceleration of the ball in the *X*, *Y*, and *Z* directions. We conducted an experiment to compare this single-network approach with the proposed method. The inputs of the single network increased to ten anterior positions so that they were consistent with the inputs of the proposed method. The input, hidden, and output layers of the model structure in the single-network approach were 40, 10, and 6. In contrast, the input, hidden, and output layers of the model structure in our dual-network approach were 40, 10, and 7, and the second model was set as 7-20-7. The both networks used the mean-square error as the loss function and the hyperbolic tangent sigmoid as the activation function. The number of epochs was 10,000. The Levenberg–Marquardt algorithm was used for optimization. Table 5 summarizes the details of the two ANNs. After applying the trained models to the testing data, we calculated the overall mean error, standard deviation, and the success rate of balls falling within the boundaries of the paddle. Figure 16 presents the mean errors of the proposed method and the single-network approach (39.6 mm and 42.9 mm), as well as their success rates (89% and 86.7%). Based on the experiment results, we can conclude that the prediction performance of the proposed method is better than that of the single-network approach. The difference between these two methods is whether they consider t he process of the ball landing point (including the landing on the table). A normal ping-pong ball trajectory consists of two trajectories in which the flight process of the ball offers data. The single-network approach only considers the data of the anterior positions and the final striking point, so its prediction performance is limited.

### 4.2. Physical Model Testing

**Figure 17:**
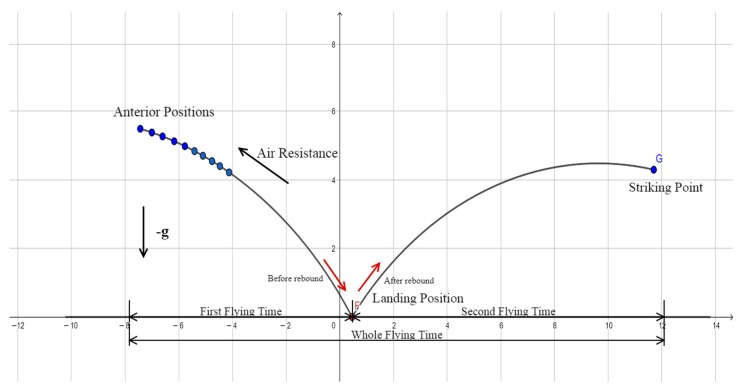
Striking point prediction using physical model.

In this section, we use a normal physical formula to predict the striking point. The method was basically the same: use the data of the ten anterior points, as shown in Figure 17, to calculate the initial velocity in three dimensions and derive the striking point while taking into account gravitational acceleration *g*, air resistance, and elastic collision. Elastic collision is associated with the coefficient of restitution of the ball, which can be obtained from real-world trajectories. For the sake of accuracy, we added 130 real-world trajectories to calculate this coefficient. The prediction process of the physical model was as follows:

1. Using the 330 trajectories, we obtained the mean velocities before and after collision and then the mean collision coefficient. Based on the formula for the coefficient of restitution in Equation (24), we concluded that the mean coefficient was 0.9203.
  (24) $$\begin{array}{c} e=\frac{\mathrm{Relative}\mathrm{velocity}\mathrm{after}\mathrm{collision}}{\mathrm{Relative}\mathrm{velocity}\mathrm{before}\mathrm{collision}} \end{array}$$
2. The *Y* and *Z* positions of the testing trajectories at *x* = 400 mm (the striking point), i.e., $Y_{true}$ and $Z_{true}$, were used to calculate the errors in the final prediction results by selecting 100 random trajectories.
3. The data from the ten anterior positions of the testing trajectories were obtained, and then the initial velocities in the *X*, *Y*, and *Z* directions, i.e., $v_{x}$, $v_{y}$, and $v_{z}$, using polynomial regression.
4. The downward accelerate $a_{z}$ was defined with air resistance taken into account as shown in Equation (25), where *g* denotes gravitational acceleration and equals 9.81 m/s^2^; *m* is the weight of the ping pong ball, which is approximately 2.7 × 10^−3^ kg; $C_{d}$ is the resistance coefficient and equals 0.5; $\rho_{a}$ denotes air fluid density, which is 1.29 kg/m^3^; and *A* is the cross-sectional area of the ball, which is roughly 1.3 × 10^−3^ m^2^. The mathematical formula indicates that the velocity and acceleration of the object change with time. Here, we set sampling time $T_{s}$ to be 0.005 s.
  (25) $$\begin{array}{c} a_{z}=g-\frac{C_{d}\cdot \rho_{a}\cdot A}{2m}v_{z}^{2} \end{array}$$
5. Using the physical formulas in Equations (26)–(28), we derived the next displacement (acceleration and velocity are updated at each sampling time).
  (26) $$\begin{array}{c} S_{z}=v_{z}T_{s}+\frac{1}{2}a_{z}T_{s}^{2} \end{array}$$
  (27) $$\begin{array}{c} v_{t+1}=v_{t}+a_{t}T_{s} \end{array}$$
  (28) $$\begin{array}{c} a_{t+1}=g+\frac{C_{d}\cdot \rho_{a}\cdot A}{2m}v_{t+1} \end{array}$$
6. When the ball reached the table (*Z* direction), we used the mean collision coefficient obtained in the first step to calculate the velocity of the ball after landing, as shown in Equation (29). Once the velocity was calculated, we could continue to calculate the position of the ball in the *Z* direction.
  (29) $$\begin{array}{c} v_{after}=v_{before}\cdot e \end{array}$$
7. Not considering the influences of friction between the ball and the table surface and the self-rotation of the ball, we could calculate the displacement of the ball in the *X* direction at each sampling time using Equations (30) and (31). The velocity and acceleration of the ball in this direction also changed with time, all the way to the striking point (*x* = 400 mm). The timing of the striking point, $T_{end}$, was then recorded.
  (30) $$\begin{array}{c} a_{x}=\frac{C_{d}\cdot \rho_{a}\cdot A}{2m}v_{x}^{2} \end{array}$$
  (31) $$\begin{array}{c} S_{x}=v_{x}T_{s}+\frac{1}{2}a_{x}T_{s}^{2} \end{array}$$
8. Finally, we used the initial velocity in the *Y* direction, $v_{y}$, air resistance acceleration, $a_{y}$ in Equation (32), and timing of striking point, $T_{end}$, to derive the striking point in the *Y* direction. Using the predicted *Y* and *Z* positions of the striking point, $Y_{predict}$ and $Z_{predict}$, and the actual striking point, $Y_{true}$ and $Z_{true}$ (Step 2), we then calculated the error using Equation (33).
  (32) $$\begin{array}{c} a_{y}=\frac{C_{d}\cdot \rho_{a}\cdot A}{2m}v_{y}^{2} \end{array}$$
  (33) $$\begin{array}{c} \mathrm{Error}=\sqrt{{\left(Y_{predict}-Y_{true}\right)}^{2}+{\left(Z_{predict}-Z_{true}\right)}^{2}} \end{array}$$

**Figure 18:**
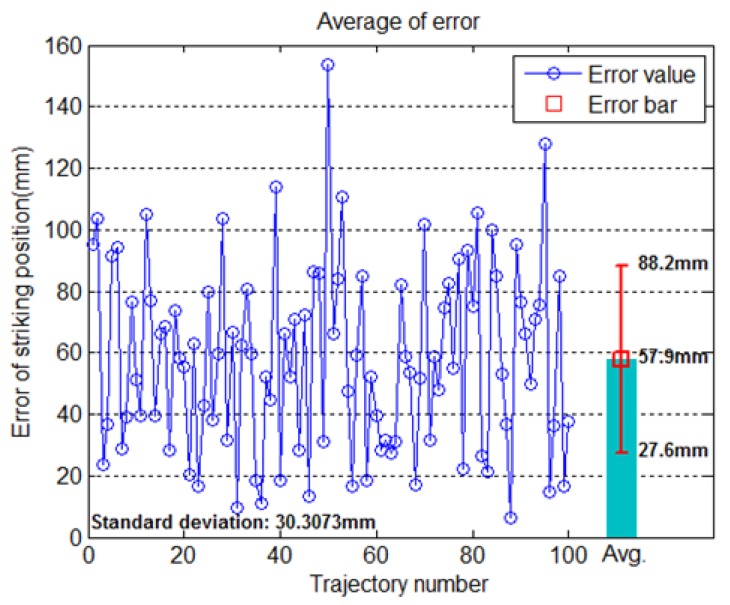
Striking position error of the physical model.

Using the physical prediction method above, we selected 30 testing trajectories to calculate striking point prediction errors. The success rate of balls hitting the paddle was 70%, and Figure 18 shows that the overall mean error and standard deviation of striking position were 57.9 mm and 30.3 mm, respectively. The success rate of 70% of the physical model was worse than 97% of the proposed dual-network method. In reality, the ball trajectory is affected by the friction of the table surface and the Magnus effect, but it is difficult to obtain precise measurements of them without more advanced vision equipment [20].

### 4.3. Removal of Time Data

**Figure 19:**
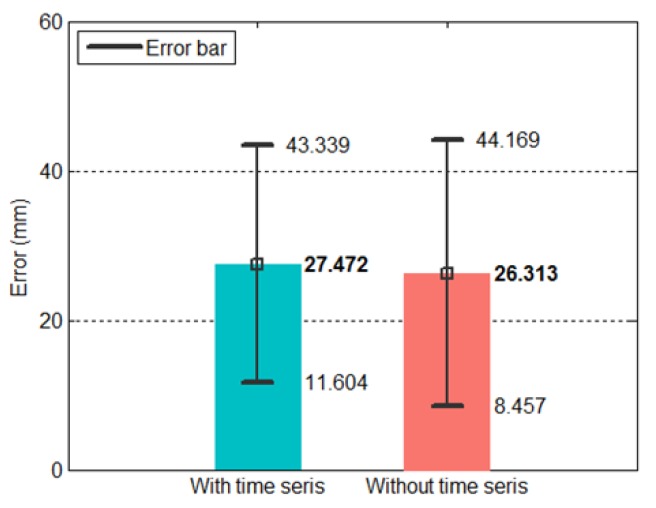
Prediction error of the proposed dual-network method with and without time stamps.

The vision system in this study had a fixed sampling time. Based on the training data, the average time interval was 0.0075 s and the standard deviation was 5.19 × 10^−4^. The inputs of the first ANN include time stamp data, which comprises steady values. For this reason, we tried removing the time stamps from the input and then tested the resulting performance. We used the data from all 330 trajectories, with 30 trajectories serving as the testing data and ten training sessions each. As shown in Figure 19, there are no significant differences between the prediction results of the two conditions. Thus, removing the time stamps from the input of the first ANN does not affect its prediction performance.

**Figure 20:**
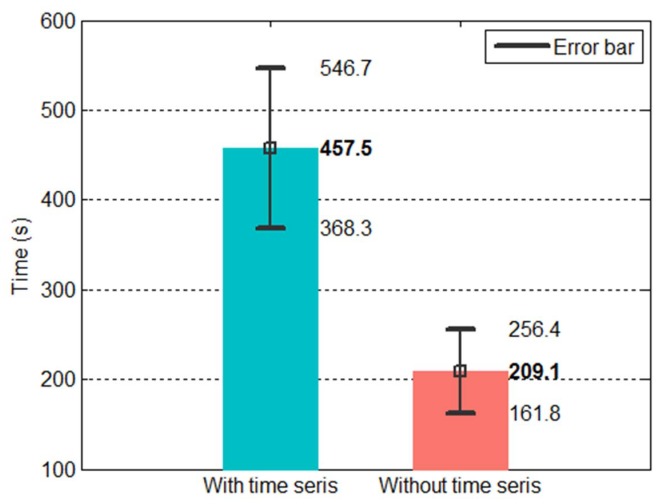
Training time of the dual-network method with and without time stamps.

This approach has its advantages. It simplifies the entire network because the inputs of the first ANN were reduced from 40 nodes to 30 nodes (see Equation (22)), which also means that the number of parameters in the first ANN decreased. The original prediction model contained a total of 1178 parameters (of which the first ANN accounted for 871 parameters), whereas the current model had 998 parameters (of which the first ANN accounted for 691 parameters). This represents a 15% reduction in the overall number of parameters. The two conditions also differed in training time. Figure 20 shows the training time of the ten tests conducted for each condition. The mean training time was 457 s for the model with the time stamps and 209 s for the model without the time stamps, representing a 54% reduction in training time.

### 4.4. Striking Point Errors

**Figure 21:**
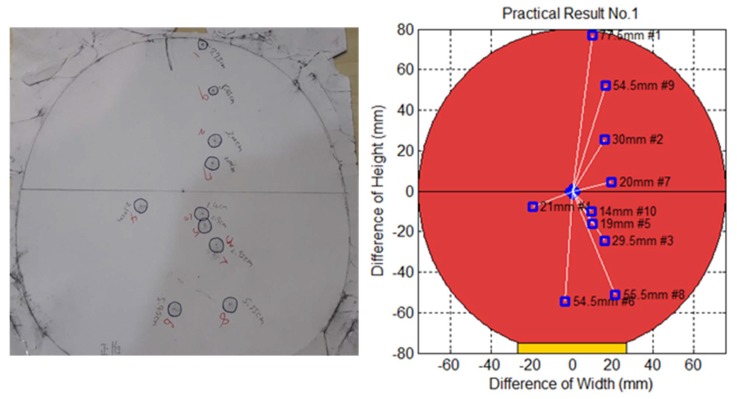
Striking points on carbon paper.

**Figure 22:**
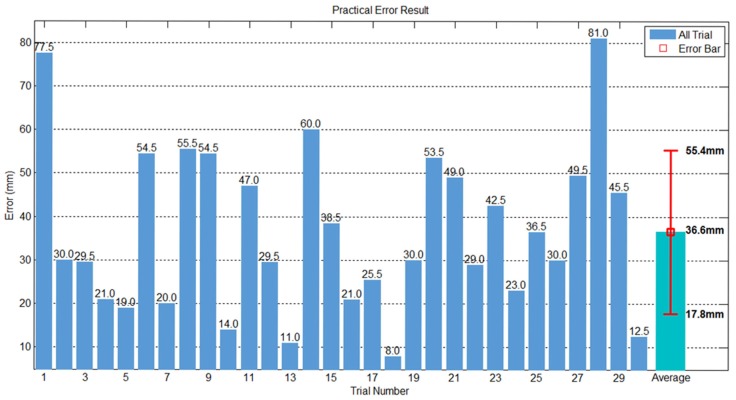
Overall striking errors.

**Figure 23:**
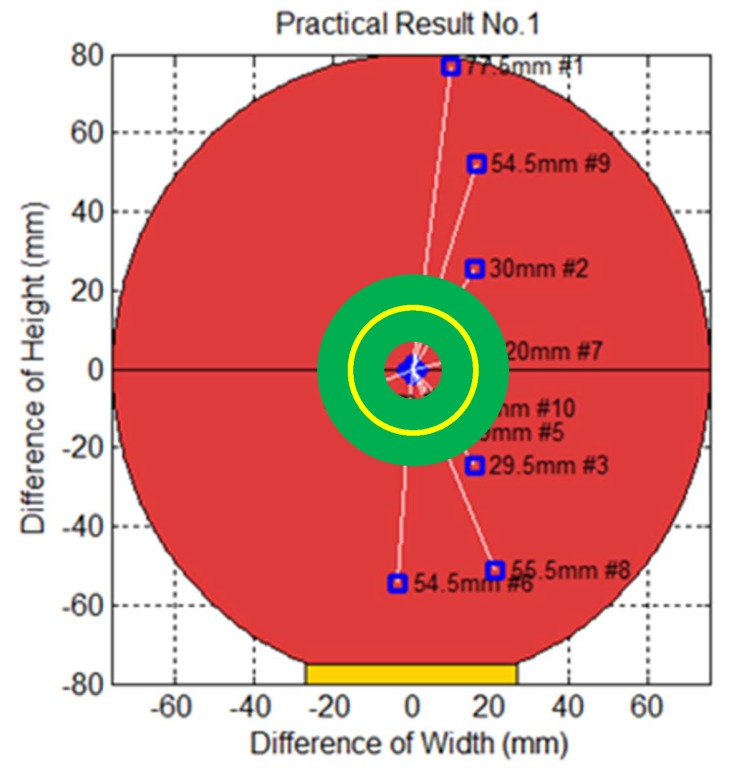
Striking accuracy with respect to the paddle area: the green area represents the area of striking error center with the mean (yellow) and a standard deviation.

To measure the prediction error of the proposed method, we conducted an experiment as follows. This experiment was done by covering the front of the paddle held by the robot arm with a piece of white paper and then covering that with a piece of carbon paper. When the robot arm struck the ping-pong ball with the paddle, the ball left a mark on the white paper, and the position of the robot arm at the time was also recorded. The distance between the center of the paddle and the striking points (block dots) indicate the prediction errors, which we measured using a vernier caliper. The area that the robot arm could strike was 20 cm × 18 cm, which equals 360 cm^2^. We conducted three sets of experiments with ten trials each, which produced a total of 30 records. Figure 21 displays the first trials of the striking points recorded on carbon paper in our physical experiment. Figure 22 shows that the thirty trials and the overall mean error was 36.6 mm, and the standard deviation was 18.8 mm. Figure 23 shows the striking accuracy with respect to the paddle area. The yellow circle represents the mean of error and the green area represents a standard deviation of error. Obviously, the area of striking error is much smaller than the paddle area, which means that the robot was able to strike the ball.

### 4.5. Experiment Discussion

In Section 4.1, we compared the striking point prediction performance of the dual-network method and a single ANN. In Section 4.2, we compared the striking point prediction performance of the physical model. Table 6 summaries the prediction error among the proposed dual-network method, single network, and physical model. The results show that the proposed method has less trajectory error than the other methods.

## 5. Conclusions

This study developed a ping-pong ball trajectory prediction system that included ball recognition, 3D positioning, and ball trajectory prediction. Trajectory prediction performance is key to whether a table-tennis robot can hit the ping pong ball. However, most existing studies developed physical models for prediction, which can only achieve good prediction effects if they have high-frequency vision systems to analyze ball status in real time. Such advanced equipment is not readily accessible and makes it difficult to conduct fundamental studies. We therefore adopted machine learning to predict the flight trajectories of ping-pong balls, which uses historical data to learn the regularities within. There is no need to establish a complex physical model, and fairly good prediction results can be achieved even with general industrial cameras readily available on the market. Each complete flight trajectory consists of a landing point on the table and two parabolic trajectories. We used two ANNs to learn the features of these flight trajectories. The first ANN learns the first flight trajectory. Once the vision system receives the anterior positions, it can instantly predict the first trajectory. We demonstrated that this approach was superior to curve fitting due to the limited amount of data and noise filtering capabilities. The second ANN learns the second flight trajectory. Once the first trajectory is known, the second flight trajectory can be instantly predicted. The two ANNs were then combined.

A comparison of the ANN and curve fitting approaches revealed that the use of data from ten anterior positions resulted in mean errors of 10.8 mm in the prediction results of the ANN and 19.2 mm in those of the quadratic polynomial resulting from curve fitting. We conducted a simulation experiment using 200 real-world trajectories as training data. The mean errors of the proposed dual-network method and a single-network model were 39.6 mm and 42.9 mm, respectively, and the mean success rates were 88.99% and 86.66%. These results indicate that the prediction performance of the proposed dual-network method is better than that of the single-network approach. We also used a simple physical model to predict striking points. We employed 330 real-world trajectories, and the resulting mean error and success rate were 57.9 mm and 70%. The success rate of 70% of the physical model was worse than 97% of the proposed dual-network method. In the proposed dual-network method, the inputs of the first ANN include time stamps. As our vision system takes samples at fixed time intervals, little variation exists in the time stamp data. We thus tried removing the time stamps from the data. We used 330 trajectories for training. The mean errors of the proposed method with and without the time stamps were 27.5 mm and 26.3 mm, and the mean success rates were 98.67% and 97.33%. The results show that even without the time stamps, the proposed method maintains its prediction performance with the additional advantages of 15% fewer parameters in the overall network and 54% shorter training time. Finally, we tested the striking ability of our robot arm, which produced a mean error and standard deviation of 36.6 mm and 18.8 mm, respectively.

## Figures and Tables

**Table 1:** Intrinsic parameters of the right camera.

	U Axis	V Axis	Error+	Error−
Focal Length (pix)	1043.25281	1049.54675	2.24798	2.12426
Principal Point (pix)	633.44849	561.99736	2.89133	2.42647
Pixel Error (pix)	0.26777	0.30308		

**Table 2:** Intrinsic parameters of the left camera.

	U Axis	V Axis	Error+	Error−
Focal Length (pix)	1049.44688	1052.73077	1.52311	1.51341
Principal Point (pix)	597.44982	513.76570	2.18344	1.66587
Pixel Error (pix)	0.23623	0.26618		

**Table 3:** Extrinsic parameters of the right camera.

	*X* Axis	*Y* Axis	*Z* Axis
Translation vector (mm)	669.420640	−371.402272	2129.031554
Rotation matrix	−0.821611	−0.568894	−0.036249
	−0.262626	0.434195	−0.861686
	0.505947	−0.698451	−0.506146
Pixel Error (pix)	0.21674	0.33150	

**Table 4:** Extrinsic parameters of the left camera.

	*X* Axis	*Y* Axis	*Z* Axis
Translation vector (mm)	−90.227708	−381.155522	2279.359066
Rotation matrix	−0.850091	0.526621	0.003993
	0.265893	0.435734	−0.859905
	−0.454585	−0.729936	−0.510438
Pixel Error (pix)	0.15424	0.32854	

**Table 5:** Details of the two ANNs.

	1st ANN	2nd ANN
Input node number	40	7
Hidden node number	10	20
Output node number	6	7
Activation fun.	Hyperbolic tangent sigmoid	Hyperbolic tangent sigmoid
Loss fun.	Mean-square error	Mean-square error
Epoch number	10,000	10,000
Optimizer	Levenberg–Marquardt	Levenberg–Marquardt

**Table 6:** Comparison of the prediction error among the proposed dual-network method, single network, and physical model.

	Proposed Dual Networks	Single Network	Physical Model
Mean (mm)	39.553	42.858	57.862
